# Supplementary material for: Osteocytes Exposed to Titanium Particles Inhibit Osteoblastic Cell Differentiation via Connexin 43
Source: Int J Mol Sci. 2023 Jun 29;24(13):10864. doi: 10.3390/ijms241310864 (PMC10342113; doi:10.3390/ijms241310864)
Supplement: Supplementary file 1 [file ijms-24-10864-s001.zip › ijms-2412624-supplementary.pdf]

Supplementary materials

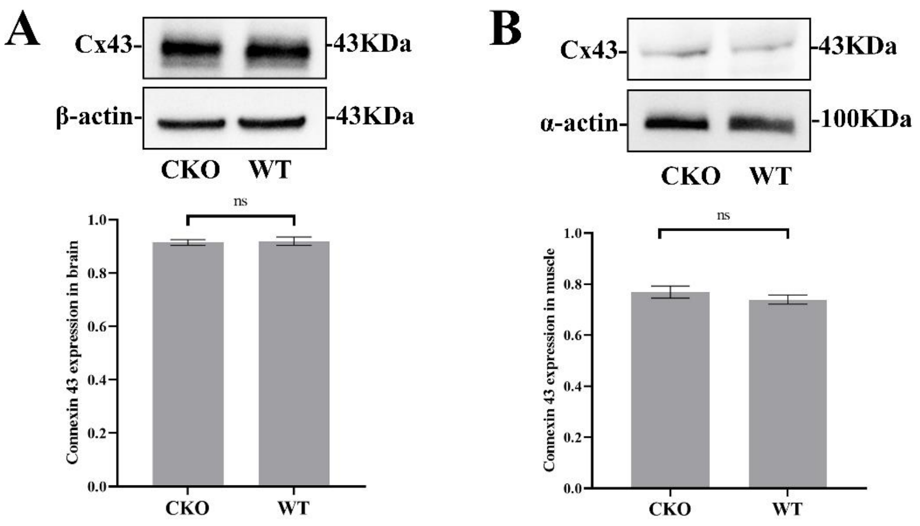

Supplementary Figure S1. Protein from brain and muscle of CKO and WT mice were extracted and the protein expression of Cx43 were determined. (A) Western blot analysis of Cx43 expression in brain. (B) Western blot analysis of Cx43 expression in muscle and α-actin was used to normalize Cx43 expression. The western blot data showed that Cx43 protein levels were not significantly different between CKO and WT mice. (CKO: Cx43-deficient mice, WT: wild type mice) Data are expressed as mean ± SD, n = 3, NS: no significant.

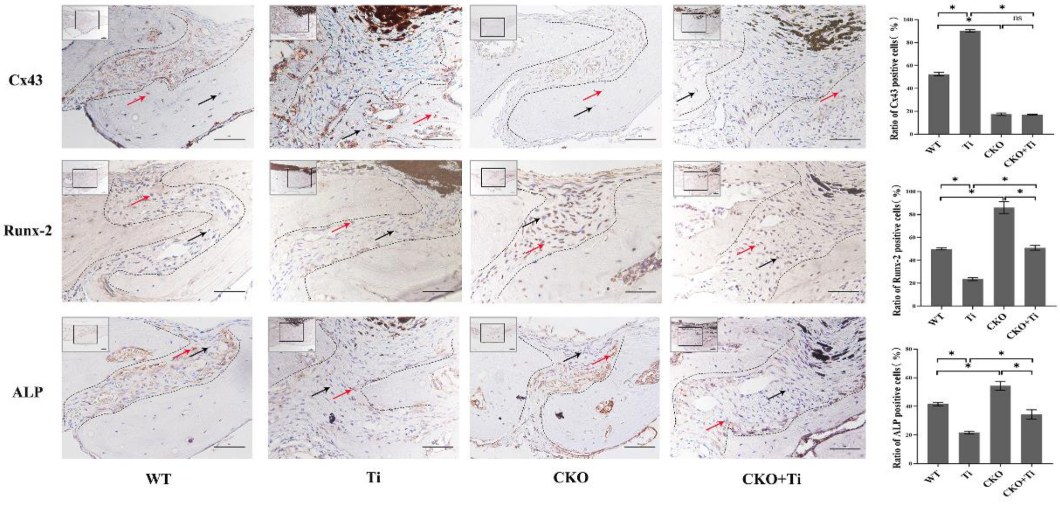

Supplementary Figure S2. Immunohistochemical stain of Cx43, Runx-2 and ALP showed representative sections were chosen from in sagittal of mouse calvarial. The black boxed regions were viewed at higher magnification. Area within the curves indicates the sagittal suture. Red arrows represent Cx43, Runx-2 and ALP positive cells, black arrows represent Cx43, Runx-2 and ALP negative cells in sagittal suture. Statistic results are displayed right, on the basis of the statistical results quantification of Cx43, Runx-2 and ALP positive cells proportion in sagittal of mouse calvarial. Three slides were selected from each group for quantitative analysis. Numbers of immunopositive cells and total cells within the sagittal suture

were counted using the Image J software. The proportion of positive cells (brown) was calculated as follow: proportion of positive cells = (the number of positive cells in sagittal / the number of total cells in sagittal) ×100%. Data are expressed as mean ± SD, n = 3, * $p < 0.05$.

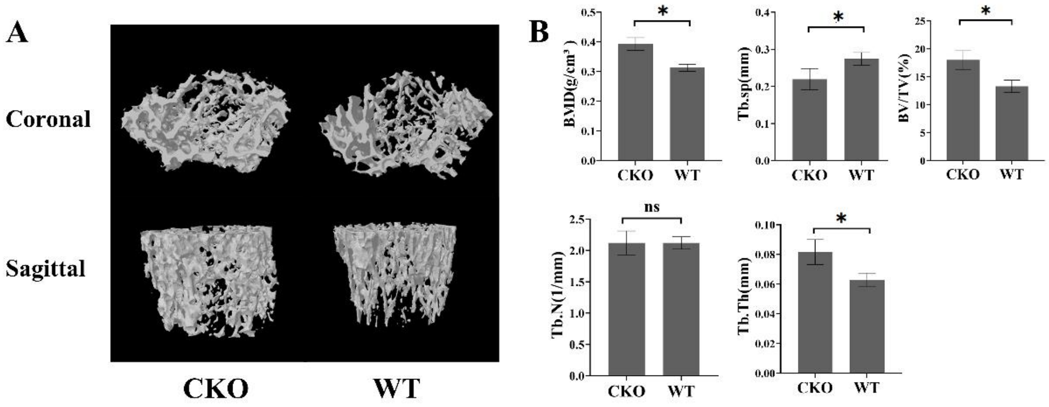

Supplementary Figure S3. Micro-CT reconstruction images of trabecular area of distal femur of CKO and WT mice, and quantitative Micro-CT analysis of area of distal femoral trabecular bone parameters (A) Coronal (above) and sagittal (below) Micro-CT images reconstruction of trabecular area of distal femur of CKO and WT mice. (B). Some parameters were analyzed using software based on micro-CT scanning data, such as BMD, BV/TV, Tb.N, Tb.Th, and Tb.SP. (CKO: Cx43-deficient mice, WT: wild type mice). Three micro-CT scan results of distal femur specimens were selected for data analyze each group. Data are expressed as mean ± SD, n = 3, * $p < 0.05$, NS: no significant.
